# The expression of SIRT1 regulates the metastaticplasticity of chondrosarcoma cells by inducing epithelial-mesenchymal transition

**DOI:** 10.1038/srep41203

**Published:** 2017-01-23

**Authors:** Helin Feng, Jin Wang, Jianfa Xu, Congcong Xie, Fulu Gao, Zhiyong Li

**Affiliations:** 1 Department of Orthopedics, The Fourth Hospital of Hebei Medical University, 12 Health Road, Shijiazhuang 050011, Hebei, People’s Republic of China; 2 Key Laboratory of Physiology and Molecular Biology of Hebei Province, College of Life Science, Hebei Normal University, 20 Nanerhuan East Road, Shijiazhuang 050024, Hebei, People’s Republic of China; 3 Department of Orthopedics, The Third Hospital of Hebei Medical University, 139 Ziqiang Road, Shijiazhuang 050051, Hebei, People’s Republic of China

## Abstract

SIRT1 belongs to the mammalian sirtuin family and plays an important role in deacetylating histone and nonhistone proteins. It is reported that SIRT1 is associated with tumor metastasis in several kinds of tumors. However, the effect of SIRT1 on the metastasis of chondrosarcoma cells is still unknown. In this study, we demonstrated that up and down-regulation of SIRT1 expression could significantly change the invasive and metastatic potential in chondrosarcoma cell line. Besides that, the result from the nude mice confirmed the effect of SIRT1 on metastasis of chondrosarcoma cells. Furthermore, we also found that SIRT1 effectively enhanced the metastasis by inducing epithelial-mesenchymal transition (EMT) in chondrosarcoma cells. Inhibition the expression of SIRT1 could block the incidence of metastasis and EMT in chondrosarcoma cells. In addition, we also observed that SIRT1 could enhance the expression of Twist which is a key transcriptional factor of EMT. A clinicopathological analysis showed that SIRT1 expression was significantly correlated with the poor prognosis of pelvis chondrosarcoma. Kaplan-Meier survival curves revealed that positive SIRT1 expression was associated with poor prognosis in patients with pelvis chondrosarcoma. Taken together, these results indicate that SIRT1 may promote the metastasis of chondrosarcoma by inducing EMT and can be a potential molecular target for chondrosarcoma therapy.

Chondrosarcoma is a malignant tumor of mesenchymal origin which generally locally aggressive and tend to produce early systemic metastases. The combination of surgical resection and combinational chemotherapy is suggested to be a regular therapies^1^. Although the long-term outcome for patients who undergo surgery for high-grade chondrosarcoma has been improved with the addition of systemic chemotherapy, prognosis remains unsatisfactory^2^. Pelvis chondrosarcoma often grows slowly and gradually, and when the tumor was found, it always relatively grown big and with metastasis. Generally resection of pelvis chondrosarcoma is the most important therapeutic modality. But surgical approaches are restricted due to the tumor size and some adjacent body structures^3^,^4^,^5^.

Sirtuins is a molecular family with seven members named from SIRT1 to SIRT7 respectively. It also shares extensive homologies in mammals with the *Sirt2* gene in yeast. Sirtuins play an important role in regulating some critical biological function in cells including metabolism, aging, oncogenesis and so on refs 2, 6. SIRT1 is a well-documented member of sirtuin family and plays a major role in controlling the survival and death of the cells by interacting with nuclear factor-κB family, p53 family members and FOXO transcription factors^7^. The exactly effect of SIRT1 in tumor development is still controversial. It has been reported that the expression of SIRT1 decreased in breast cancer^8^. However, SIRT1 expression is upregulated significantly in several cancer such as leukemia, lymphomas, prostate cancer, colon carcinoma and lung cancer^9^,^10^,^11^,^12^,^13^. The promotive effect of SIRT1 on tumor metastasis was also reported in hepatocellular carcinoma^14^,^15^.

In this study, we observed the potentialeffect of SIRT1 on regulating metastasis in chondrosarcoma cells *in vitro* and *in vivo*. We also detected the underlying mechanism of SIRT1 in regulating chondrosarcoma cells metastasis. The correlations among SIRT1 expression, and survival time of patients with pelvis chondrosarcoma were assessed.

## Materials and Methods

### Cell Culture

Human chondrosarcoma cell line SW1353 cells and HS.819.T cells were cultured in Dulbecco’s modified Eagle medium (DMEM; Invitrogen Gibco Cell Culture Products, Carlsbad, CA) supplemented with 10% fetal bovine serum (FBS; Invitrogen, Carlsbad, CA), 50 units of penicillin 50 μg/mL gentamicin, 2.5 μg/mL amphotericin B, 1% glutamine, 2% HEPES at 37 °C in a humidified atmosphere containing 5% CO_2_.

### The construction of SIRT1-recombinant adenovirus vector and cell transfection

According to the manufacturer’s instruction, the recombined adenoviral expression vectors were constructed by the Gateway Cloning System (Invitrogen, Carlsbad, CA, USA). The sequences of shRNA were designed by the Oligoengine software.

SW1353 cells and HS.819.T cells (1 × 10^6^ respectively) were cultured in 10 cm petri dishes to 60% confluence and then were transfected with adenoviral vectors by using the Lipofectamine 2000 kit (Invitrogen, cat. 11668-019) according to the manufacturer’s procedure. Then the cells were harvested 48 h after transfection and transfection efficiency was examined by western blot.

### Wound healing and Transwell assays

The methods for wound healing and the transwell assay have been described^16^,^17^. The distance migrated bythe cell monolayer to close the wounded area during this time period was measured. Results were expressed as a migration index—that is, the distance migrated by treated cells relative to the distance migrated by control. Experiments were carried out in triplicate.

Cell migration assay was performed using Transwell (Costar, Corning Life Sciences, Acton, MA; pore size, 8 μm) in 24-well plate. About 1 × 10^4^ SW1353 cells in 200 μL of serum-free medium containing were placed in the upper chamber, and 300 μL of the same medium was placed in the lower chamber. The plate was incubated at 37 °C in a humidified atmosphere containing 5% CO_2_ for 48 hours. At the end of the experiment, the cells that passed through the filters were stained with crystal violet solution and counted under a microscope. These experiments were performed in triplicate.

### Cell Counting Kit-8(CCK-8) assay

The proliferation of the cells was assessed by CCK-8 (Dojindo, Japan). The Cells were cultured in 96-well plates (5 × 10^3^ cells/well). After 72 h, CCK-8 (10 μl solution) was added in each well. Then the plate was incubated for in a humidified CO_2_ incubator further 2 hours at 37 °C. Finally, the absorbance of each well was examined by spectrophotometer (Synergy HT, Bio-Tek) at 450 nm.

### Nude mouse splenic vein metastasis assay

All experimental procedures involving animals were performed in accordance with the institutional ethical guidelines from the Animal Ethics Committee of the Hebei Medical University. Nude mice were then anesthetized by inhalation of diethyl ether, and the spleen was exteriorized through a flank incision. SW1353 cells (5 × 10^5^/300 μl/mouse) were slowly injected in 8-week-old nude mice via the splenic vein through a 27-gauge needle and the spleen was then removed. The mice were sacrificed after 6 weeks, the livers were harvested and liver metastasis nodule were measured^18^.

### RNA extraction and real-time quantitative PCR

Total RNA extraction, complementary DNA (cDNA) synthesis, and qPCR were performed as described previously^19^. The SW1353 cells were extracted the total mRNA with RNeasy Mini Kit (Qiagen, Valencia, CA) according to the manufacturer’s protocol. One microgram of total RNA was used to generate the first strand cDNA using random primers and SuperScript II reverse transcriptase (Invitrogen). Real-time PCR was performed in triplicate using the SYBR PrimeScript RT-PCR Kit (Takara, Dalian). The expression of GAPDH was measured as an internal control. Thermocycler conditions included an initial hold at 50 °C for 2 minutes and then 95 °C for 10 minutes; then followed a two-step PCR program of 95 °C for 15 seconds and 60 °C for 60 seconds repeated for 40 cycles on an Mx4000 system (Stratagene, La Jolla, CA), on which data were collected and quantitatively analyzed. Expression level of mRNA was demonstrated as fold change relative to the control group. The primer sequences used in the qPCR were shown in Table 1.

### Protein extraction and Western blotting analysis

Extraction of total soluble proteins from cultivated cells and western-blot analysis were performed as previously described^17^. Primary antibodies used in western blot analysis that were specific for SIRT1 (1:100, Santa cruz, Inc), and Twist (1:2000, Novus Biologicals, Inc) were incubated overnight with the membranes at 4 °C. Horseradish peroxidase conjugated goat anti-rabbit IgG was used as the secondary antibody (R&D systems, Cat. BAF008). GAPDH was used as the internal control to normalize the loading materials.

### Immunohistochemical staining and scoring

Immunohistochemistry was performed according to the IHC-P (immunohistochemistry-paraffin) staining protocol of Abcam (Cambridge, UK), the specimens were fixed in 4% paraformaldehyde and embedded in paraffin. Sections (4 μm) were incubated with anti-SIRT1 (1:100 dilution, Santa Cruz) mouse anti-human monoclonal antibodies, then with multiuse secondary antibody (1:1000 dilution, Dako, UK). Staining was visualized with an EnVisionTM Peroxidase/DAB Rabbit/Mouse detection kit (Dako, UK). Immunostaining results were independently evaluated by two clinical pathologists with no knowledge of the clinicopathological features. In the event of differing evaluations, a final decision was made by consensus. A sample scored for strong SIRT1 expression in previous studies was used as a positive control, and primary antibodies were replaced with PBS for a negative control. Five high-power fields were randomly selected for each sample. Immunoreactivity was categorized into five semi-quantitative classes depending on the percentage of stained cancer cells: 0 (negative), 1 (1–10% positive cells), 2 (11–50% positive cells), 3 (51–75% positive cells), and 4 (>75% positive cells). The immunostaining intensity was also determined semi-quantitatively by light microscopy on a scale of 0–3 as follows: 0 (negative), 1 (weakly positive), 2 (moderately positive), and 3 (strongly positive). According to the Remmele-Scoring system, the final staining score was graded using a combination of the intensity and the percentage scores: negative (0), +(1–4), ++(5–8), and +++(9–12). For statistical analysis, tumors scored negative or + were classified as low expression and tumors scored ++ classified as moderate, +++ were classified as high expression.

### Immunofluorescence

SW1353 cells (1 × 10^4^/well) were seeded in a 48-well dish. After 24 hours, the cells were washed in PBS twice and fixed in 3.7% formaldehyde for 30 min at room temperature, permeabilized with 0.2% Triton X-100 for 5 min at room temperature and blocked with PBS containing 3% bovine serum albumin (BSA). After blocking, cells were simultaneously incubated with primary antibodies for 1 h at room temperature. After washing with PBS, cells were incubated with secondary antibodies for 1 hour at room temperature. Cell nuclei were stained with DAPI. All matched samples were photographed with immunofluorescence microscope.

### Patients and samples

The present study evaluated paraffin-embedded specimens (34 pelvis chondrosarcoma) that were collected between 2000 and 2011 from patients attending the Fourth Hospital of Hebei Medical University. Clinical and histopathological characteristics and follow-up and survival information were available for all patients, and were collected retrospectively from medical records. Patients data were grouped according to age, sex, tumor size (<10 cm vs. ≥10 cm), recurrence (positive vs negative), distant metastasis and pathological type (Table 2). All patients had undergone pelvis chondrosarcoma resection. This study was approved by the Human Ethics and Research Ethics Committees of the Fourth Hospital of Hebei Medical University. All patients provided written informed consent. We confirm that all methods were performed in accordance with the relevant guidelines and regulations. The end of follow-up was defined as the date of death or last contact up to June 2015. Overall survival (OS) was defined as the time from the date of diagnosis to the date of last contact or death. Patients who were alive at last contact were censored for OS analysis.

### Statistical analysis

Each experiment was carried out at least three times with consistent results. The representative gel or blot for each experiment is presented in this study. The correlation between SIRT1 and individual clinicopathologic parameters was evaluated using the nonparametric chisquare test. The Kaplan-Meier method was used to estimate survival rates for SIRT1 expression. Equivalences of the survival curves were tested by log-rank statistics. The Kaplan-Meier method was used to illustrate the OS further. All statistical analyses were conducted using SPSS software (version 21.0), and *p* < *0.05* was considered statistically significant.

## Results

### Regulating SIRT1 expression changed the metastatic potential in human chondrosarcoma cells

**Figure 1:**
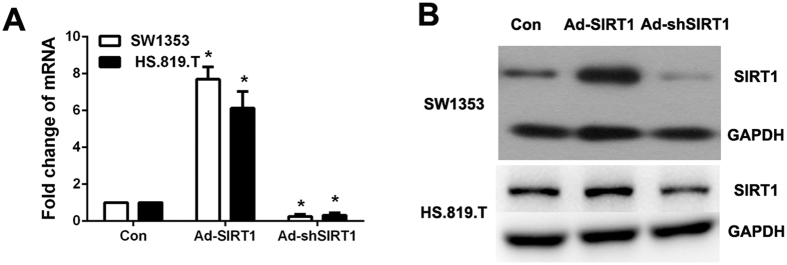
The expression of SIRT1 in SW1353 and HS.819.T cells and transfection efficiency of adenovirus vectors. (**A**) and (**B**) The expression of SIRT1 in SW1353 and HS.819.T cells, and transfection efficiency of adenovirus vectors including Ad-SIRT1 and Ad-shSIRT1 was examined by realtime PCR and western blot.

Firstly, we examined the SIRT1 expression and the efficiency of the adenovirus vectors that we used to regulate SIRT1 expression in human chondrosarcoma cells, SW1353 and HS.819.T cell line. We constructed adenovirus-mediated over expressed SIRT1 and shRNA knockdown to elucidate the cellular functions at the protein level. As shown in Fig. 1A and B, SIRT1 was spontaneously expressed in SW1353 and HS.819.T cells and the adenovirus vectors that we used to regulate SIRT1 expression could effectively up and down-regulate the expression of SIRT1 in SW1353 and HS.819.T cells.

**Figure 2:**
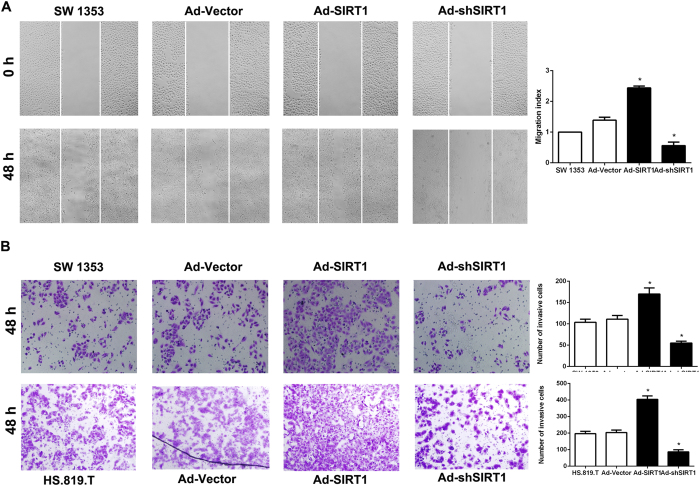
Regulating SIRT1 expression changed the metastatic potential in human chondrosarcoma cells. (**A**) Wound healing assay was used to detect the migration of SW1353 cells. The cells were plated in 6-well plate and the cells were monitored every 24 hours to observe the migration rate in the scratched area (×200; *P < 0.05). (**B**) Invasion of SW1353 and HS.819.T cells was examined by transwell assay. The cells were cultured in the upper chamber of the transwell for 48 hours in serum-free medium, while 5% fetal bovine serum was placed in the lower chamber. The number of cells which has invaded into matrigel was counted in 10 random fields under microscope (×200; *P < 0.05).

Next, we observed the effect of SIRT1 on the migration and invasion of SW1353 and HS.819.T cells. We employed wound-healing and transwell assay to detect the role of SIRT1 on the cell migration and invasion. We also used CCK-8 assay to examine the effect of SIRT1 on the proliferation of the cells. As shown in supplementary Fig. 1, up or down-regulating the expression SIRT1 in SW1353 cells did not obviously affect the proliferation of cells. However, it could be observed that SIRT1 (Ad-SIRT1) over expression in cells could significantly enhance the cell migration and invasion, whereas SIRT1 (shSIRT1) depletion markedly diminished wound-healing capacity and impaired cell invasion through transwell matrigel (Fig. 2A and B).

These results indicate that SIRT1 plays an important role in regulating migration and invasion of chondrosarcoma cells *in vitro*.

### The role of SIRT1 in regulating chondrosarcoma cells metastasis *in vivo*

**Figure 3:**
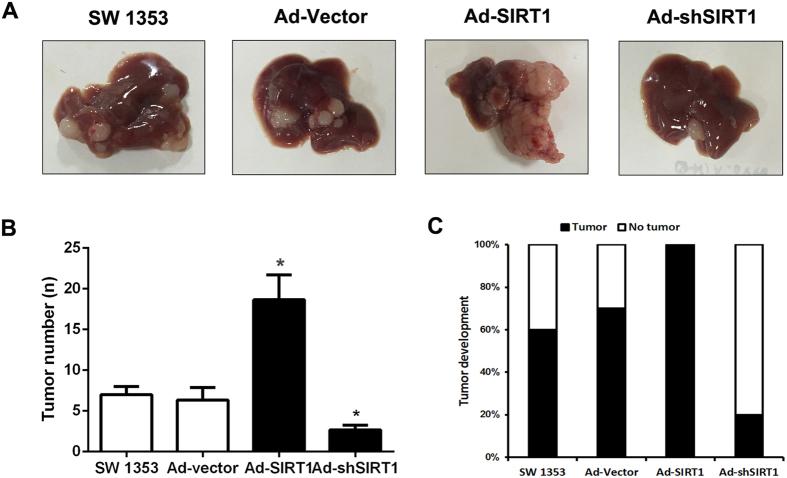
The role of SIRT1 in regulating chondrosarcoma cells metastasis *in vivo*. (**A**) The Ad-SIRT1 and Ad-shSIRT1 was transfected respectively into SW1353 cells and then the cells (1 × 10^5^/mouse) were injected in nude mice via splenic-vein. (**B**) The number of tumor was quantified in the livers (n = 10 per group). (**C**) The tumor occurrence rate was quantified in the livers (n = 10 per group).

We have known that SIRT1 can effectively regulate the migration and invasion of chondrosarcoma cells *in vitro*. Then we explored the effect of SIRT1 on the chondrosarcoma cells metastasis *in vivo*. We employed nude mouse splenic vein metastasis assay to examine the metastatic potential of SW1353 cells *in vivo*. We injected the SW1353 cells that were transfected with Ad-SIRT1 or sh-SIRT1 into splenic vein of each nude mouse. Six weeks later, the nude mice in each group were sacrificed and the metastatic nodules on the liver were observed and evaluated. As shown in Fig. 3A,B and C, The number and incidence rate of metastatic nodules in the liver increased significantly in the Ad-SIRT1 group and decreased in the sh-SIRT1 group compared with control group. These results indicate that SIRT1 has effects on the metastatic potential of SW1353 cells *in vivo*.

### Regulation the SIRT1 expression in chondrosarcoma cells leads to the change of epithelial-to-mesenchymal transition level

**Figure 4:**
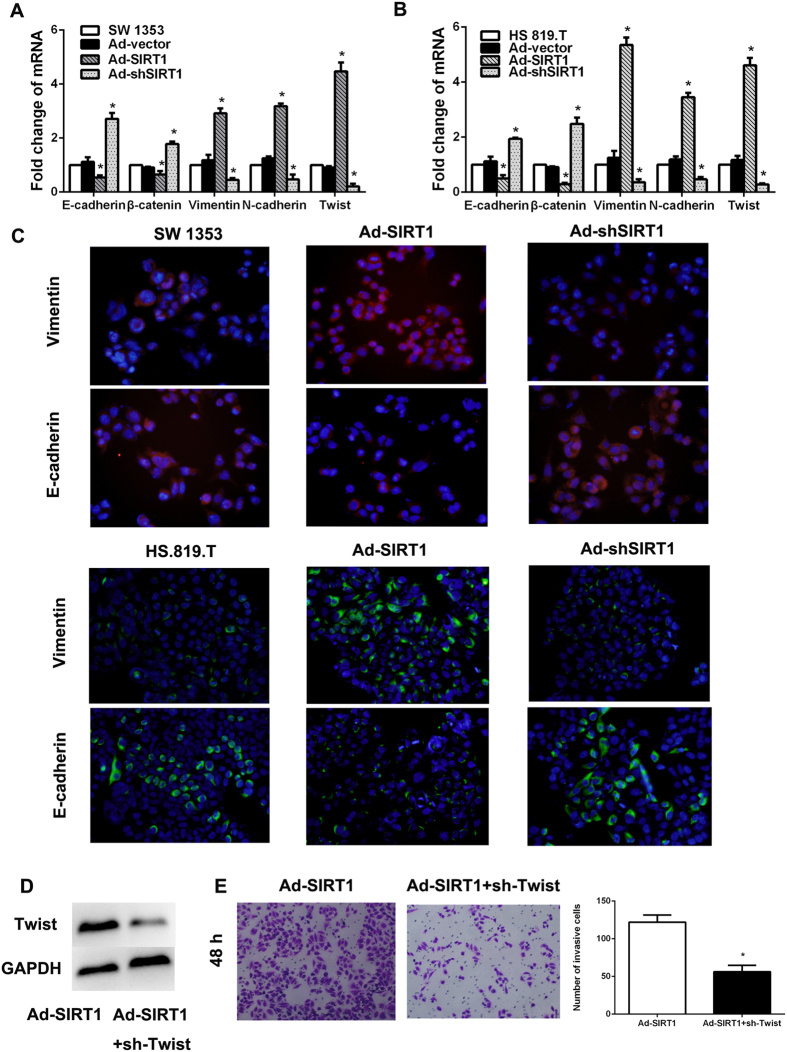
Regulation the SIRT1 expression in chondrosarcoma cells leads to the change of epithelial-to-mesenchymal transition level. (**A** and **B**) SW1353 and HS.819. T cells (1 × 10^6^/well) were cultured in 6-well plate for 24 h with the existence of Ad-SIRT1 or Ad-shSIRT1. Then the cells were collected and realtime PCR was employed to examine the expression of EMT associated genes including Vimentin, N-cadherin, Twist, E-cadherin and β-catenin. (**C**) Immunofluorescent staining of E-cadherin and Vimentin was performed in SW1353 HS.819.T cells, nuclei were counterstained with DAPI. The pictures were captured by fluorescence microscopy (×200). (**D**) Western blot was performed to detected Twist expression. (**E**) The Twist in SW1353 cells was inhibited by shRNA and then the metastasis potential of the cells was examined by transwell assay. The cells were cultured in the upper chamber of the transwell for 48 hours in serum-free medium, while 5% fetal bovine serum was placed in the lower chamber. The number of cells which has invaded into matrigel was counted in 10 random fields under microscope (×200; *P < 0.05).

It has been reported that EMT played a key role in the metastasis of tumor cells^20^. In order to explore the potential mechanism that SIRT1 regulate the migration and invasion of chondrosarcoma cells, we performed RT-PCR and immunofluorescence staining to examine EMT-associated markers in SW1353 and HS.819.T cells. The results demonstrated that overexpression SIRT1 could effectively lead to the up-regulation of the mesenchymal markers (Vimentin and N-cadherin) and down-regulation of the epithelial markers (E-cadherin and β-catenin) (Fig. 4A and B). However, when the SIRT1 expression in SW1353 and HS.819.T cells was down-regulated by sh-SIRT1, the expression of EMT associated factors demonstrated a reversal tendency compared with Ad-SIRT1 group (Fig. 4A and B). The same results were seen according to immunofluorescence data (Fig. 4C). On one hand, these results suggested that SIRT1 expression could effectively control the EMT level of chondrosarcoma cells. On the other hand, the results indicated that the change of EMT level in chondrosarcoma cells reflected the dramatic plasticity of chondrosarcoma cells.

It has been proved that the incidence of EMT in tumor cells is regulated by several transcription factors, such as Twist^21^. So we examined the Twist expression in SW1353 cells which has been treated with Ad-SIRT1 or sh-SIRT1. As shown in Fig. 4A, Ad-SIRT1 or sh-SIRT1 could significantly lead to up or down-regulation of Twist respectively. Besides, inhibition the Twist expression could significantly decrease the metastasis potential in SW1353 cells which could be increased by Ad-SIRT1 (Fig. 4D and E).

Taken together, these results indicated that SIRT1 might regulate the metastatic plasticity of chondrosarcoma cells by inducing EMT.

### The correlation between SIRT1 expression and the prognosis of pelvis chondrosarcoma

**Figure 5:**
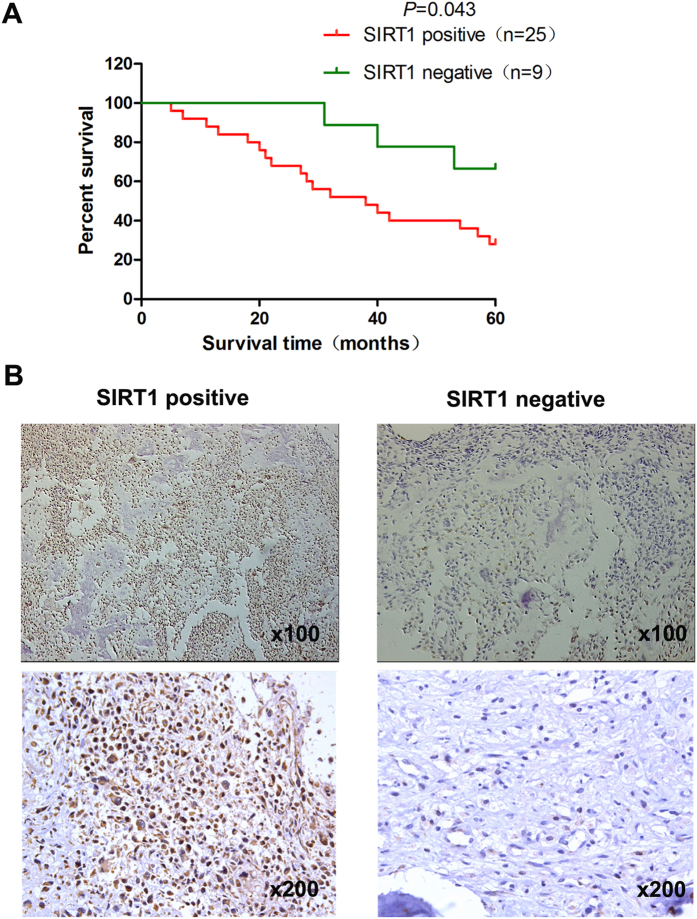
The correlation between SIRT1 expression and the progression and prognosis of pelvis chondrosarcoma. Immunohistochemical analysis of SIRT1 expression in chondrosarcoma. (**B**) Nuclear expression of SIRT1 (×200) was negative in 9/34 of pelvis chondrosarcoma tissues, and positive in 25/34. The patients with chondrosarcoma were divided into positive- and negative-expression groups based on SIRT1 immunostaining scores. The survival rate of the patients in the positive-expression group was significantly lower than that of patients in the negative-expression group (**A**) (P = 0.043 by the log-rank test).

We first examined SIRT1 expression levels in clinical pelvis chondrosarcoma specimens. Immunohistochemical staining results showed that SIRT1 was overexpressed in pelvis chondrosarcoma. SIRT1 overexpression was observed in 25 of 34 (73.5%) pelvis chondrosarcoma specimens. All 34 patients with pelvis chondrosarcoma were divided into a positive expression group (n = 25) and a negative expression group (n = 9) (Fig. 5B). Significant associations were observed between SIRT1 expression and the size of tumor, recurrence, metastasis and pathological type (*P* = 0.004, *P* = 0.008, *P* = 0.023, *P* = 0.002, respectively) for the 34 patients with pelvis chondrosarcoma. Relationships between SIRT1 expression and clinicopathological features of the patients with pelvis chondrosarcoma are summarized in Table 2. Metastases developed in 19 patients among which 12 patients developed local recurrence. Sixteen cases were lung metastasis and three cases were multiple metastases. In addition, patients with pelvis chondrosarcoma in the negative SIRT1 expression group showed a favorable survival outcome (*P* = 0.043), with mean survival times of 53.8 months, respectively compared with those of the positive expression group survival times of 37.7 months. The SIRT1 expression survival curves in tumors are shown in Fig. 5A.

## Discussion

Sirtuins is a molecular family with seven members named from SIRT1 to SIRT7 respectively. It also shares extensive homologies in mammals with the Sirt2 gene in yeast. It has been reported that the relationship of SIRT1 expression and tumor metastasis in several kinds of tumors. In this study, we demonstrated that up and down-regulation of SIRT1 expression could significantly lead to the change of invasive and metastatic potential in chondrosarcoma cell line. Besides, the result from the nude mice confirmed the effect of SIRT1 on metastasis of chondrosarcoma. However, the tumor metastasis includes several different steps and the nude mouse splenic vein metastasis assay that we used in this study only mimicpart of the migration. Furthermore, we also found that SIRT1 effectively enhanced the metastasis by inducing EMT in chondrosarcoma cells. Inhibition the expression of SIRT1 could inhibit the incidence of metastasis and EMT in chondrosarcoma cells. In addition, we also observed that SIRT1 could enhance the expression Twist which is a key transcriptional factor of EMT instead of Snail. A clinicopathological analysis showed that SIRT1 expression was significantly correlated with pelvis chondrosarcoma. Kaplan-Meier survival curves revealed that positive SIRT1 expression was associated with poor prognosis in patients with pelvis chondrosarcoma.

SIRT1 plays an important role in regulating several biological functions, such as metabolism, aging, DNA damage and tumor development^22^. The expression of SIRT1 is upregulated in prostate cancer, acute myeloid leukemia, and primary colon cancer^13^,^23^,^24^. However, it has been reported that SIRT1 expression decreased in several kinds of cancers, including ovarian cancer, human bladder and glioblastoma^25^. So the role of SIRT1 in tumorigenes is is still controversial. In our study, we found that regulating the expression of SIRT1 in chondrosarcoma cells will lead to the change of the metastatic plasticity of the cells. Then high expression of SIRT1 in chondrosarcoma is always correlated with a poor prognosis in pelvis chondrosarcoma patients.

EMT is a process by which the mesenchymal phenotype is acquired by epithelial cells, which is a key process in a variety of human epithelial tumors. EMT is characterized by loss of polarity and cell-cell contacts in epithelial cells followed by a dramatic remodeling of the cytoskeleton^20^. The incidence of EMT in tumor cells always indicates the enhancement of tumor metastasis^26^,^27^. These findings strengthen the hypothesis that SIRT1 acts as a tumor promoter.

Our results demonstrated that the expression of SIRT1 in chondrosarcoma cells could effectively regulate the metastatic plasticity of the cells by inducing epithelial-mesenchymal transition. Furthermore we also observed that SIRT1 could enhance the expression of Twist which is a key transcriptional factor of EMT instead of Snail. Besides that we also found the correlation between SIRT1 expression and the progression and prognosis of chondrosarcoma patients. These findings provide information that will facilitate development of a novel therapeutic approach against chondrosarcoma metastasis.

In conclusion, our results indicate that SIRT1 may promote the metastasis of chondrosarcoma cells by inducing EMT and can be a potential molecular target for chondrosarcoma therapy.

## Additional Information

**How to cite this article**: Feng, H. *et al*. The expression of SIRT1 regulates the metastaticplasticity of chondrosarcoma cells by inducing epithelial-mesenchymal transition. *Sci. Rep.* **7**, 41203; doi: 10.1038/srep41203 (2017).

**Publisher's note:** Springer Nature remains neutral with regard to jurisdictional claims in published maps and institutional affiliations.

## Supplementary Material

Supplementary Dataset 1

## Figures and Tables

**Table 1:** Sequences of RT-PCR oligonucleotide primers.

Gene	Sequence (5′ → 3′)	
E-cadherin	F	TGAAGGTGACAGAGCCTCTGGA
	R	TGGGTGAATTCGGGCTTGTT
Vimentin	F	TGGCCGACGCCATCAACACC
	R	CACCTCGACGCGGGCTTTGT
Twist	F	GCCAGGTACATCGACTTCCTCT
	R	TCCATCCTCCAGACCGAGAAGG
SIRT1	F	TGCTGGCCTAATAGAGTGGCA
	R	CTCAGCGCCATGGAAAATGT
GAPDH	F	TGCCAAATATGATGACATCAAGAA
	R	GGAGTGGGTGTCGCTGTTG

**Table 2:** Relationship between SIRT1 expression and the clinicopathological features of 34 patients with pelvis chondrosarcoma.

Parameters	Category	Total no. of patients	SIRT1 positive (n = 25)	SIRT1 negative (n = 9)	*P*
Age (years)	>40	19	13 (52.0)	6 (66.7)	0.360
	≤40	15	12 (48.0)	3 (33.3)	
Gender	Male	19	14 (56.0)	5 (55.6)	0.640
	Female	15	11 (44.0)	4 (44.4)	
Size of tumor (cm)*	≥10	22	20 (80.0)	2 (22.2)	0.004
	<10	12	5 (20.0)	7 (77.8)	
Recurrence*	Positive	17	16(64.0)	1 (11.1)	0.008
	Negative	17	9 (36.0)	8 (88.9)	
Metastasis*	Positive	19	17 (68.0)	2 (22.2)	0.023
	Negative	15	8 (32.0)	7 (77.8)	
Grade*	I	15	7 (28.0)	8 (88.9)	0.002
	II + III	19	18 (72.0)	1 (11.1)	
