# Supplementary material for: The expression of SIRT1 regulates the metastaticplasticity of chondrosarcoma cells by inducing epithelial-mesenchymal transition
Source: Sci Rep. 2017 Jan 23;7:41203. doi: 10.1038/srep41203 (PMC5255567; doi:10.1038/srep41203)
Supplement: Supplementary Dataset 1 [file srep41203-s1.pdf]

The expression of SIRT1 regulates the metastaticplasticity of chondrosarcoma cells by inducing epithelial-mesenchymal transition

Helin Feng, Jin Wang, Jianfa Xu, Congcong Xie, Fulu Gao, Zhiyong Li

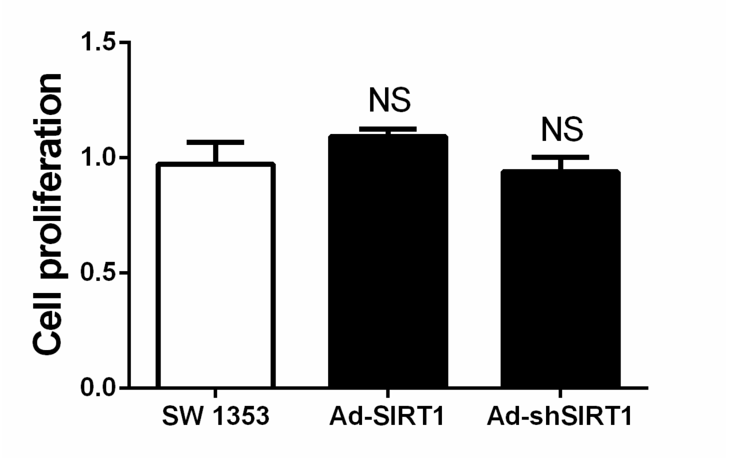
Cell proliferation
1.5
1.0
0.5
0.0
NS
NS
SW 1353
Ad-SIRT1
Ad-shSIRT1
